# An in-silico approach to develop of a multi-epitope vaccine candidate against SARS-CoV-2 envelope (E) protein

**DOI:** 10.21203/rs.3.rs-30374/v1

**Published:** 2020-05-20

**Authors:** Fatemeh Ghafouri, Reza Ahangari Cohan, Farshid Noorbakhsh, Hilda Samimi, Vahid Haghpanah

**Affiliations:** Shahid Beheshti University; Pasteur Institute of Iran; Tehran University of Medical Sciences; Tehran University of Medical Sciences; Tehran University of Medical Sciences

**Keywords:** SARS-CoV-2, Envelope (E) protein, COVID-19 vaccine, In-silico

## Abstract

Since the first appearance of the Severe Acute Respiratory Syndrome Coronavirus 2 (SARS- CoV-2) in China on December 2019, the world has now witnessed the emergence of the SARS- CoV-2 outbreak. Therefore, due to the high transmissibility rate of virus, there is an urgent need to design and develop vaccines against SARS-CoV-2 to prevent more cases affected by the virus. In this study, a computational approach is proposed for vaccine design against the envelope (E) protein of SARS-CoV-2, which contains a conserved sequence feature. First, we sought to gain potential B-cell and T-cell epitopes for vaccine designing against SARS-CoV-2. Second, we attempted to develop a multi-epitope vaccine. Immune targeting of such epitopes could theoretically provide defense against SARS-CoV-2. Finally, we evaluated the affinity of the vaccine to major histocompatibility complex (MHC) molecules to stimulate the immune system response to this vaccine. We also identified a collection of B-cell and T-cell epitopes derived from E proteins that correspond identically to SARS-CoV-2 E proteins. The in-silico design of our potential vaccine against E protein of SARS-CoV-2 demonstrated a high affinity to MHC molecules, and it can be a candidate to make a protection against this pandemic event.

## Introduction

The recent outbreak of the new virus in Wuhan City, China, originated possibly from the seafood industry has contributed to the discovery of a new strain named coronavirus and labeled the SARS-CoV-2 of the Coronaviridae family. This virus has caused severe damage and anxiety, leading to the loss of myriad individuals, impacting more than 1,000,000 people (https://who.sprinklr.com/). Symptoms such as the flu-like illness, acute respiratory distress syndrome, and clinical or radiological evidence of pneumonia in individuals needing hospitalization are considered as COVID-19^1^. Patients diagnosed with COVID-19 are reported to have high levels of interleukin 1 beta (IL1B), interferon gamma (IFNγ), interferon-inducible protein 10 (IP10), and monocyte chemoattractant protein 1 (MCP1), likely leading to activated T-helper-1 (TH1) cell responses. In comparison, patients needing ICU admission had higher concentrations of granulocyte-colony stimulating factor (GCSF), IP10, MCP1, MIP1A, and tumor necrosis factor α (TNFα) than those not needing, suggesting a possible correlation of cytokine storm and disease intensity. Nonetheless, SARS-CoV-2 infection also resulted in the enhanced production of T-helper-2 (TH2) cytokines, such as interleukin 4 (IL4) and interleukin 10 (IL10) that inhibit inflammation that varies from that of SARS-CoV infection^2^. The persistent rise in patients and the high contagious rate of SARS-CoV-2 infection illustrate the immediate need to develop a safe and effective vaccine.

Vaccines were mostly made up of whole pathogens, either destroyed or attenuated. It may be beneficial to use peptide vaccines that are capable of generating an immune response against a specific pathogen^3^. Epitope-based vaccines (EVs) utilize immunogenic peptides (epitopes) to cause an immune response. The performance of the EV is calculated by the number of epitopes to be used as the foundation. Nevertheless, the experimental identification of the nominee epitopes is costly in terms of time and money. Besides, different immunological requirements need to be considered for the final choice of epitopes^4^.

**Figure 1:**
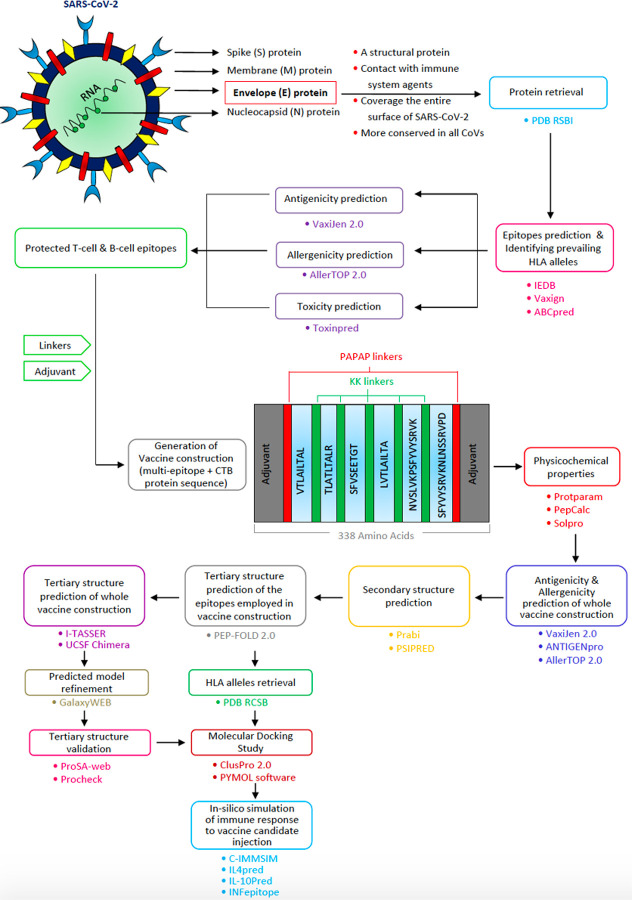
From Envelope (E) protein of SARS-CoV-2 to multi-epitope vaccine Schematic description and the study work flow of in-silico design a multi-epitope vaccine against envelope (E) protein of SARS-CoV-2

The electron microscope determines the properties of CoVs. They are enveloped viruses with single-stranded positive-sense RNA. The coronavirus genome size varies from 26–32 kb^5^. Like all coronaviruses, SARS-CoV-2 comprises four viral proteins, namely spike (S) protein, a form of glycoprotein, membrane (M) protein, covering the membrane, envelope (E) protein, a strongly hydrophobic protein that covers the entire coronavirus structure, and nucleocapsid (N) protein, a structural protein that suppresses the RNA interference (RNAi) in order to overcome the host defense^6,7^ (Fig. 1). Such accessory proteins are not only essential for virion assembly but could also play an extra function in disrupting the host immune responses to promote viral replication^8^. Structural proteins spike and membrane are shown to have substantial mutational modifications, while envelope and nucleocapsid proteins (Fig. 1) are highly conserved indicating differential selection pressures imposed on SARS-CoV-2 during evolution^9^. Envelope (E) protein is a small intrinsic membrane protein actively engaged in several stages of the life cycle of the virus, such as assembling, propagation, enveloping, and pathogenesis^10^. This protein also slows the transport of proteins through the secretive pathway by adjusting the concentrations of Ca^2+^ and H^+^ in the Golgi and endoplasmic reticulum (ER) compartments and has been suggested to be a mechanism for immune avoidance^11^. The envelope (E) protein sequence was collected from a protein database and analyzed with various bioinformatics tools to identify protective epitopes. The toxicity of whole protein was analyzed, and nine epitopes including TALRLCAYCC, ALRLCAYCCN, LRLCAYCCNI, RLCAYCCNIV, LCAYCCNIVN, CAYCCNIVNV, AYCCNIVNVS, YCCNIVNVSL, and CCNIVNVSLV were identified as toxic epitopes. The predicted B-cell and T-cell epitopes were checked in terms of not coinciding with these regions. The toxic epitopes of envelope (E) protein were less than the spike protein. This finding was verified in the literature in which envelope (E) protein was explored in Severe Acute Respiratory Syndrome (SARS) in 2003 and, more recently, in the Middle East Respiratory Syndrome (MERS), the retention of this protein against the seven strains was investigated and confirmed using BioEdit Package tool^10,12–17^. Several experiments have explored the ability of CoVs with mutated envelope (E) protein or without mutated envelope (E) protein, concentrating mainly on SARS and MERS-CoV, as live attenuated vaccine candidates with some impressive results^18–21^. Therefore, we decided to design an effective vaccine against this protein.

## Results

### Selection of protein sequences

The amino acid sequence of envelope (E) protein of SARS-CoV-2 was collected from the NCBI virus with an accession number of QHD43418 released on 2020-01-13. The FASTA sequence was used to construct a multi-epitope vaccine against SARS-CoV-2.

The sequence of “cholera toxin B (CTB) subunit [Vibrio mimicus]” was collected from the NCBI database and was used as an adjuvant.

### B-cell epitopes analysis

ABCpred database has studied the full-length E protein sequence for the analysis of linear B-cell epitopes. All results passed three more filters (antigenicity, allergenicity, and toxicity) and two epitopes (**NVSLVKPSFYVYSRVK – YVYSRVKNLNSSRVPD**) were chosen as protective epitopes (Tab 1).

### T-cell epitopes analysis

The first group of T-cell epitope prediction was performed using vaxign server (Tab 1). The next two groups including binding epitopes to MHC class I and class II molecules were predicted individually by the IEDB database. The three filters aforementioned were applied to identify the protective antigens. Based on the number of alleles, the predominant human leukocyte antigen (HLA) alleles were HLA-A*01:01, HLA-A*24:02, HLA-A*11:01, HLA-A*03:01, and HLA-B*07:02 between MHC class I alleles and HLA-DRA-DBR1*01:01 and HLA-DPB1*01:02 in MHC class II alleles.

### Antigenicity of potential epitopes

Antigenicity of both B-cell and T-cell epitopes was foreseen by VaxiJen 2.0, with a threshold of 0.4. Except two epitopes, the antigenicity score of other predicted epitopes were upper the threshold and were considered as “antigen” epitopes. The two more screenings (including allergenicity and toxicity) were not carried out on “non-antigen” epitopes (Tab 1).

### Antigenicity of potential epitopes

Allergenicity of both B-cell and T-cell epitopes was estimated by AllerTOP v. 2.0 (Tab 1).

### Toxicity of potential epitopes

Toxicity of both B-cell and T-cell epitopes was anticipated by ToxinPred, with a peptide fragment length of 10 (Tab 1).

### Construction of chimeric peptide

The tick marked epitopes in Table 1 that remained after screening were chosen for the design of chimeric peptide as a multi-epitope vaccine. Finally, we selected 12 epitopes (including 2 B-cell epitopes, 7 binding epitopes to MHC class I proteins, and 5 binding epitopes to MHC class II proteins) and they were all “antigen”, “non-allergen”, and “non-toxin”. In this part, to make a contiguous sequence in final construction, the overlapping of B-cell and T-cell epitopes were merged. Predicted linear B-cell epitopes and T-cell epitopes were connected utilizing KK linkers as flexible connectors. The “cholera toxin B subunit [Vibrio mimicus]” with GenBank accession AJP16764.1 was selected as an adjuvant and attached to the amino terminals of the multi- epitope vaccine through PAPAP linkers as rigid linkers to improve antigen-specific immune responses^22^ (Fig. 1).

### Antigenicity, allergenicity and toxicity estimation of the nominee multi-epitope vaccine

The final peptide chimera (Fig. 1) antigenicity (along with the adjuvant sequence) was estimated by the VaxiJen 2.0 server to be 0.5841 with a threshold of 0.4. The primary multi-epitope vaccine sequence (without adjuvant) reported a score of 0.6906. ANTIGENpro was another platform that was utilized to estimate the antigenicity of final peptide. Based on this server the whole protein (Fig. 1) is antigen with a probability of 0.809160.

AllerTOP v. 2.0 server estimated the allergenicity of the final peptide. The result for both states was “nonallergen”. By utilizing ToxinPred server, the final peptide was predicted as “non- toxon”.

### Amino acid composition and physicochemical properties and solubility prediction

Based on the Protparam database, the final peptide chimera comprised 338 amino acids (Fig. 1) with a molecular weight of 37.7 kDa. The isoelectric point (PI) value was expected to be 9.62. The half-life was estimated to be 30 hours, with adjuvant and 100 hours, without adjuvant in mammalian reticulocytes *in vitro* and more than 20 hours in yeast and over 10 hours in Escherichia coli (E.coli) *in vivo*. An instability index II was predicted to be 41.34, classifying the protein as unstable (II >40 indicates instability), but the amount of this index is really near the border. The estimated Grand Hydropathic Average (GRAVY) was −0.120. The negative attribute indicates that the protein is hydrophilic and can react with water molecules^22^. Furthermore, based on the PepCalc server, the solubility was predicted to be “good” in water. Based on SOLpro from ANTIGENpro, Peptide chimera was expected to be SOLUBLE with a possibility of 0.684306.

**Figure 2:**
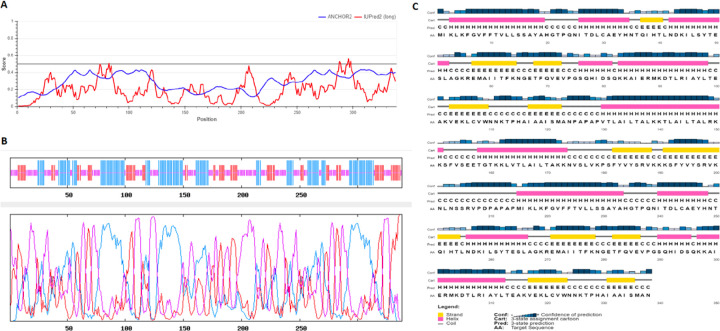
Analyzing the properties of the final peptide (A) Two parts in adjuvant regions are disorders of the final protein, which make it unstable. (B) The Prabi server analyzed the secondary structure of the final protein. Red: extended strands, Blue: Alpha helix configurations, Purple: random coils. (C) The residues and their arrangements analyzed by the PSIPRED 4.0 server. Yellow and pink regions as well as gray linkers demonstrate strands, helix, and coil configurations, respectively

By using the Iupred2a server, the disorder regions of the final peptide, which make it unstable, were identified. The disorders are regarded in the adjuvant areas (Fig. 2A).

### Secondary structure prediction

The secondary structure of the final protein (Fig. 1) was analyzed by the Prabi server, the final chimeric peptide was estimated to include 36.09% alpha-helix, 22.49% extended strand, and 41.42% random coil. There is no compactness in alpha-helix locations, which demonstrates there will be less difficulty in future synthesis steps (Fig. 2B). The other server we used to predict the secondary structure of the peptide chimera was PSIPRED 4.0 server. The results and the details of residues and their configurations are given in Figure 2C.

### Tertiary structure homology modeling

The final protein tertiary structure was built by I-TASSER server. I-TASSER provided the top 10 proteins from PDB library that had the closest structural similarity to the predicted vaccine model. The average TM-score of these ten proteins was calculated to be 0.41. We selected the best predicted model according to C-score; a confidence score to estimating the quality of predicted models by I-TASSER. It had the highest C-score of −3.96, and the RMSD was estimated to be 16.4±3.0Å.

### Tertiary structure refinement

The GalaxyWEB server was used to refine the predicted model. After refinement, an evident improvement was observed in the percentage of residues in Ramachandran favored regions relative to the initial predicted model.

### Tertiary structure validation

**Figure 3:**
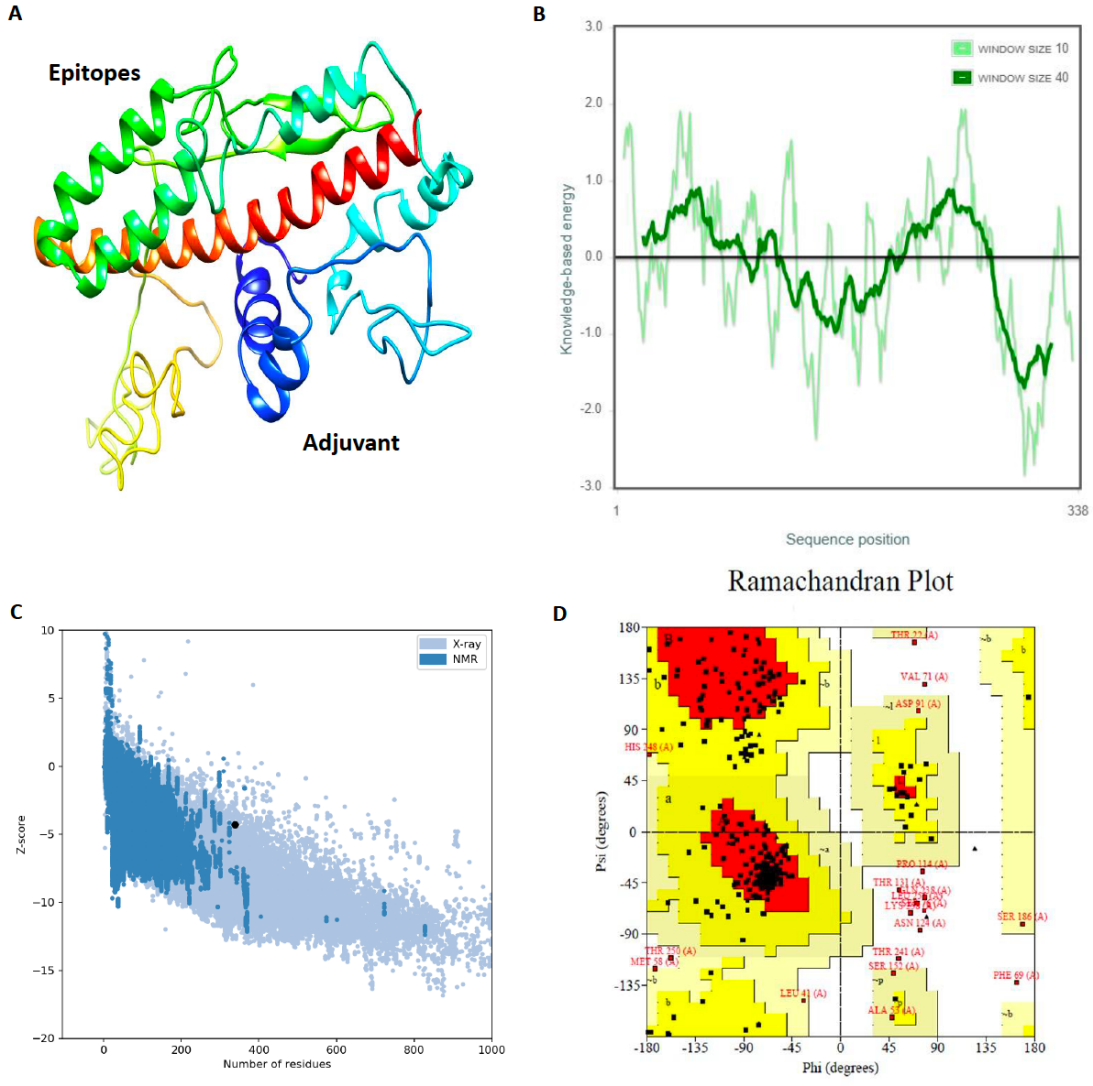
Homology modeling and validation of 3D structure (A) The final protein tertiary structure was built by the I-TASSER server. This presentation of the predicted model was performed by Chimera visualization tool. (B) The local quality model of predicted model. (C) Based on the ProSA-web, the Z-score of predicted model is −4.3 (D) The Ramachandran plot of predicted model

The plot B in Figure 3 reveals local quality model by displaying the energy as a function of the amino acid sequence location. This plot is made by ProSA-web server. In general, the negative values refer to the problematic or erroneous areas of the input model, which is observed in some parts in adjuvant regions. About our model, these negative values were observed in middle parts including epitope but the lowest value was relevant to the C-terminus connected adjuvant, not the multi-epitope section.

Based on the ProSA-web, the Z-score of peptide chimera was predicted to be −4.3 (Fig. 3C). This value is in the range of native conformations^23^.The diagram of the predicted local similarity to target and the Z-score value of the homology model is shown in Figure 3B and C.

Based on the Procheck server, The Ramachandran plot analysis of the modeled protein reported that 72.0% of residues in the protein are in the favored regions. 22.2% of the residues were predicted to be in additional allowed regions and 2.3% were in generously allowed regions, with 9 residues (3.5%) in the disallowed regions (Fig. 3D).

### Molecular docking of subunit vaccine with MHC molecules

**Figure 4:**
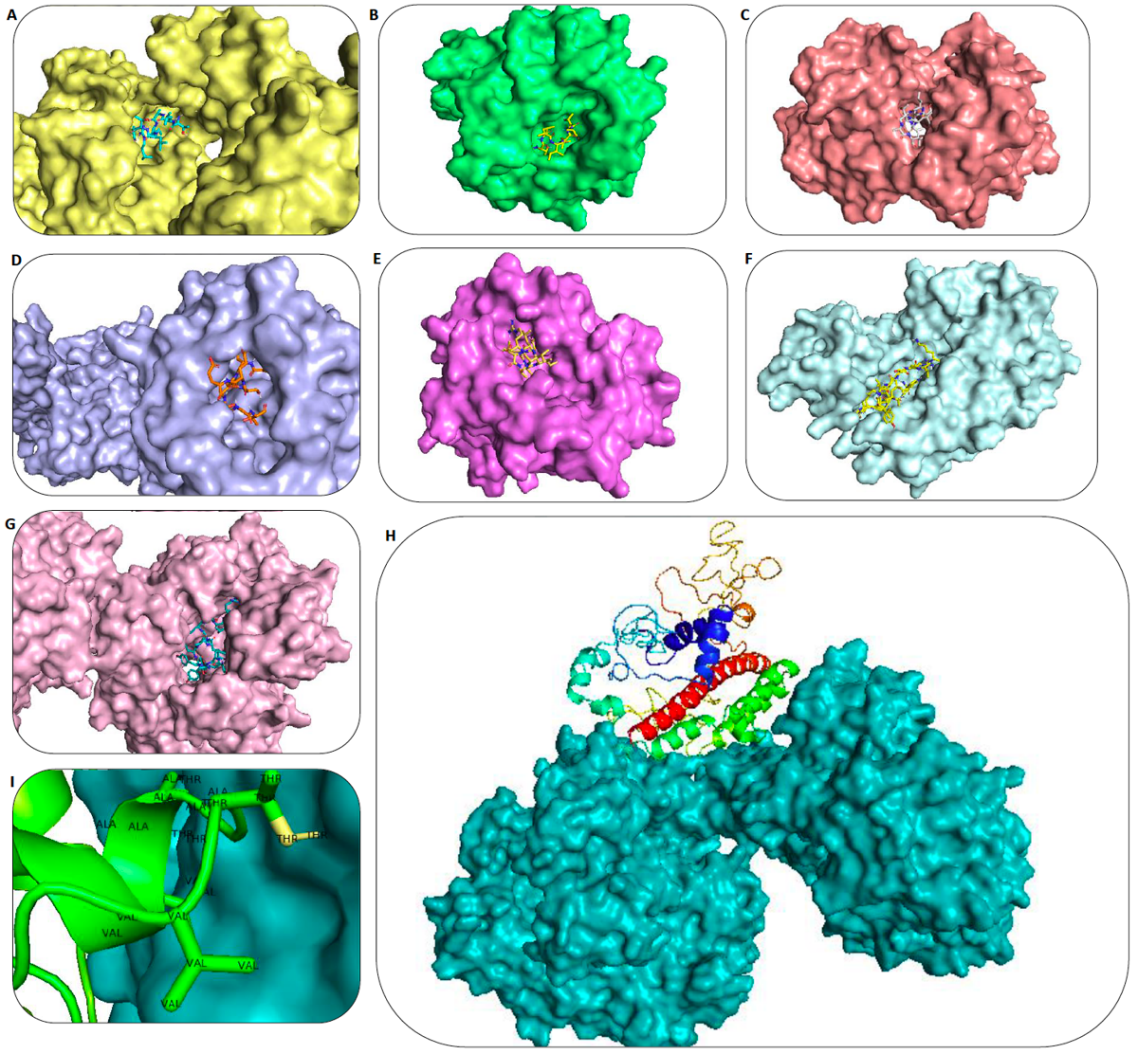
Molecular docking studies (A) TLAILTALR epitope (blue) and HLA-A*01:01 protein (cream). (B) LVTLAILTA epitope (yellow) and HLA-A*24:02 protein (green). (C) VTLAILTAL epitope (gray) and HLA-B*07:02 protein (pink). (D) SFVSEETGT epitope (orange) and HLA-A*11:01 protein (blue). (E) TLAILTALR epitope (yellow) and HLA-A*03:01 protein (purple). (F) NVSLVKPSFYVYSRVK epitope (yellow) and HLA-DPA1*01:03 protein (light blue). (G) SFYVYSRVKNLNSSRVPD epitope (blue) and HLA-DRB1*01:01protein (pink) (H) Whole vaccine peptide (colorful) and HLA-DRB1*01:01(teal blue) (I) The active residues (green) of the vaccine peptide in docked complex with HLA-DRB1*01:01 (teal blue).

Crystal structure of HLA-A*01:01, HLA-A*24:02, HLA-A*11:01, HLA-A*03:01, HLA- B*07:02, HLA-DRADBR1*01:01 and HLA-DPB1*01:02 were retrieved from the PDB RCSB database (PDB ID: 4NQX, 5HGH, 6ID4, 6O9B, 5EO0, 1AQD, 3LQZ, respectively). The PDB les were edited and cleaned from heteroatoms. PEP-FOLD2.0 from the RPBS Web Portal server was used to predict the tertiary structure of 6 epitopes of vaccine construction individually. A molecular docking study was carried out on epitopes and the whole vaccine construction with MHC alleles by the ClusPro 2.0 online server. PyMOL software was used to perform a detailed analysis of the interface of protein-protein interaction (PPI) (Fig. 4). The weighted score of the lowest energy docked complexes are reported in Table 2. The best way to rank the model is the cluster size (number of members)^24,25^.The most populated clusters were found in SFVSEETGT and HLA-A*24:02, TLAILTALR and HLA-A*24:02, VTLAILTAL and HLA-A*24:02, and TLAILTALR and HLA-A*01:01 with 997, 997, 990, and 769 cluster members, respectively.

The docking study on the whole vaccine construction with the MHC molecules depicted one of the most probable position and orientation of the peptide within the MHC binding grooves (Fig. 4H). The active residues of Fig. 4I belongs to TLAILTALR epitope which interacts with chain E of HLA-DRB1*01:01.

### Immune response simulation

We predicted the IL4, IL10 and INFγ inducing peptides from the 6 epitopes in the final vaccine construction via IL4pred server, IL10pred server and INFepitope server, respectively. The result predicted the SFVSEETGT epitope as an IL4 inducer and the NVSLVKPSFYVYSRVK as an IL4, IL10, and INFγ inducer and the SFYVYSRVKNLNSSRVPD as an IL4 and IL10 inducer peptide.

**Figure 5:**
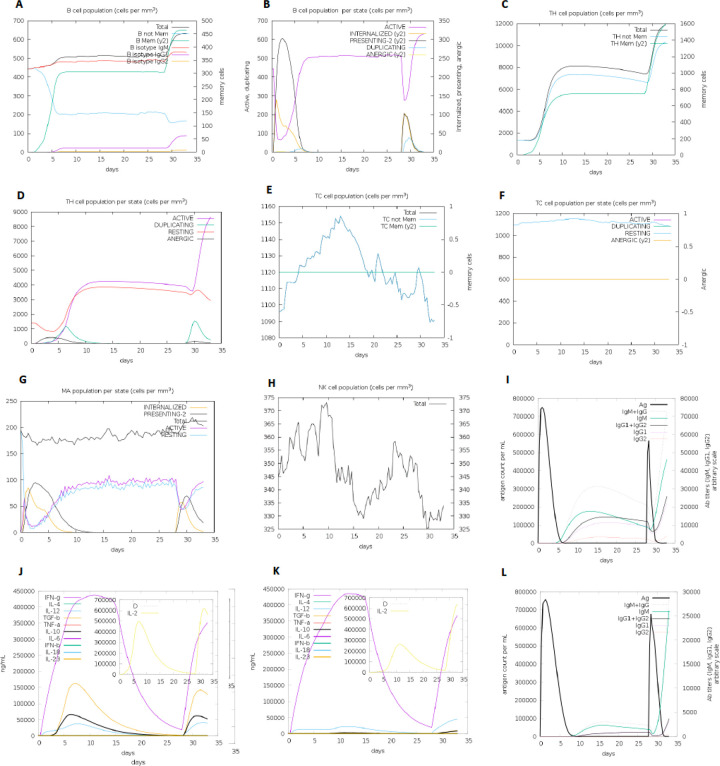
In-silico immune response simulation to the injection of candidate vaccine (without adjuvants) and control protein by C-IMMSIM server The simulation is performed for three times of injection in the time steps of 1, 84, and 100. Each time step is equal to 8 hours. (A) B-cell population. (B) B-cell population per state. (C) T helper (TH) cell population. (D) TH cell population per state. (E) T cytotoxic (TC) cell population. (F) TC cell population per state. (G) Macrophage (MA) cell population. (H) Natural killer (NK) cell population. (I) Immunoglobulins. (J) Cytokines. (K) Cytokines after the protein control injection. (L) Immunoglobulins following the protein control injection

**Figure 6:**
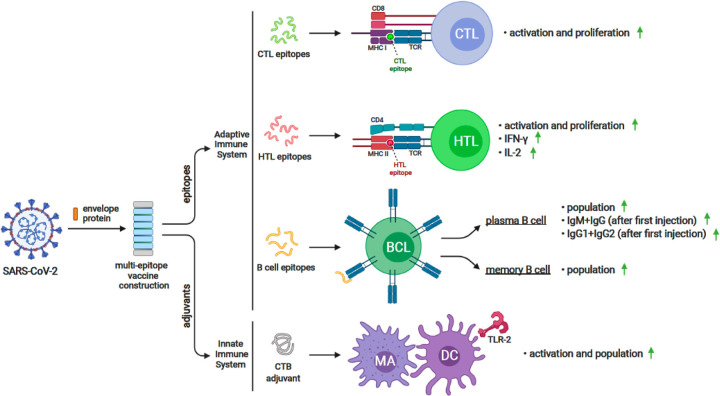
Graphical feature of immune response simulation to the injection of vaccine candidate against E protein of SARS-CoV-2 CTL: cytotoxic T lymphocyte cell, HTL: helper T lymphocyte, BCL: B-cell lymphoma, MA: Macrophage cell, DC: Dendritic cell, TLR-2: Toll Like Receptor 2, IgM: Immunoglobulin M, IgG: Immunoglobulin G, INF-γ: Interferon gamma, IL-2: Interleukin-2, CTB: cholera toxin B subunit, TCR: T-cell receptor, MHC I: major histocompatibility complex class I,, MHC II: major histocompatibility complex class II, CD4: cluster of differentiation 4, CD8: cluster of differentiation 8 The image was created by using www.biorender.com illustrator tool.

The primary and secondary immune responses were stimulated by the C-IMMSIM server. This server simulated the immune response to vaccine candidate (without adjuvants) for three times of injection in the time steps of 1, 84, and 100. Each time step is equal to 8 hours. To make a relative comparison, we made a shuffled sequence of the vaccine candidate as a control protein, and we analyzed the results of the immune response simulation to injection of it. This shuffled sequence was employed to evaluate the significance of the vaccine sequence results, because in immune response simulation by this server, the sequence composition (the final epitopes connected via KK linkers) is an important consideration. Finally, we found out that the results of the vaccine injection varied from those of the controls. The results of the immune response simulations are given in Figures 5 and 6.

## Discussion

Based on the World Health Organization (WHO) website (https://who.sprinklr.com/) as of April 13, 2020, the number of COVID-19 confirmed cases is estimated at 1,776,867 people and 111,828 deaths. Therefore, there is an immediate need to develop vaccines against this transmissible disease. There is currently no vaccination or licensed medication for humans against SARS-CoV-2. Nonetheless, further clinical trials are still needed to confirm their efficacy and safety^26^.

Epitope-based vaccines offer a new strategy for the prophylactic and therapeutic use of pathogen-specific immunity^27^. A multi-epitope vaccine consisting of a series of or overlapping peptides seems to be an appropriate solution to the prevention and treatment of viral infections^12–17^. The perfect multi-epitope vaccine should be engineered to include epitopes which can activate cytotoxic T lymphocyte (CTL), T-cells and B-cells and trigger successful responses to specific viruses^12^.

In this study, we present an in-silico design of a potential multi-epitope vaccine against the E protein of SARS-CoV-2, which is made of both B-cell and T-cell epitopes which can stimulate the immune system responses impressively. Envelope (E) protein is conserved in all CoVs and covers the entire surface of SARS-CoV-2 (Fig.1). It has less toxic regions, rather the spike protein. Several studies have examined the potential of CoVs with mutated envelope (E) protein or without mutated envelope (E) protein, focusing specifically on SARS-and MERS-CoV, as live attenuated vaccine candidates associated with hopeful results^10,18,19,28–30^. First of all, we obtained the FASTA sequence of E protein of SARS-CoV-2 from the NCBI database. B-cell and T-cell epitopes of this protein were predicted by different servers. The epitopes were screened based on three filters of antigenicity, allergenicity, and toxicity. Therefore, we selected the protective epitopes. We merged the overlaps of B-cell and T-cell epitopes and fused them by appropriate flexible linkers. Previous studies reported that KK linkers preserve independent immune responses when they are inserted between epitopes^31^. Then, we linked CTB adjuvants at the terminus of epitopes by PAPAP linkers as rigid linkers to enhance the biological activities^32^ (Fig. 1). Another study suggested that CTB, though an important adjuvant through the nasal and oral routes of administration, can also be considered to improve the immune response in intramuscular dosing vaccine regimens^33^. Immunological adjuvants are agents that improve the intensity, activation, or longevity of antigen-specific immune responses if used in conjunction with particular vaccine antigens^34^.

The absence of allergenic properties of the proposed peptide chimera further increases its potential as a vaccine candidate^22^. Finally, the whole peptide chimera was analyzed for antigenicity, allergenicity, and toxicity, and it was predicted as Antigen^35^, Non-Allergen^36^, and Non-Toxin^37^. PI was calculated to be 9.62, which shows that the final protein is alkaline. It was predicted as “soluble” upon expression in the E.coli host. The instability index II was about 1 unit over the threshold of 40, and it resulted in considering this protein as “unstable”. However, more analysis demonstrated that the residues which are responsible for such disorders to make it unstable are in adjuvant regions not in the multi-epitope area (Fig. 2A). Secondary structure analysis predicted that the final protein is consisted of 36.09% alpha-helix, 22.49% extended strand, and 41.42% random coil. Essential types of “structural antigens” have been identified as natively unfolded protein regions and alpha-helical coils peptides. These two structural types, when examined in synthetic peptides, have the capacity to fold into their native structure and are therefore recognized by antibodies naturally triggered in response to infection^22,38^. Protein three dimensional (3D) structures offer useful insights into their molecular activity and provide a wide variety of applications in bioscience^39^. The I-TASSER server modeled the tertiary structure of the final protein. Based on the Ramachandran plot, 96% of the residues of refined predicted model were found in favored and allowed regions with 3.5% outliers. Successful refining might improve the applicability of template-based models by offering more reliable structures for functional analysis, molecular design or experimental structure determination^40^. In the context of structural vaccinology, a molecular docking study was needed to forecast the binding affinity of epitopes to the crystallized fragment (FC) of antibodies or MHC molecules^41,42^. To analyze the affinity of the final multi-epitope vaccine to MHC molecules, we did 26 docking studies. They were carried out on the 6 epitopes of the final vaccine and the whole vaccine construction with MHC class I and class II receptors. The results of docking studies were notable and demonstrated the high affinity of the multi-epitope vaccine and its individual epitopes to MHC molecules. Then the interface of protein-protein interactions was reconsidered by a visualizations tool. At the next step of designing a multi-epitope vaccine, following an approach of systems vaccinology is beneficial in assessing the human complex immune response at different stages of biological structures^43^. Finally, we utilized an immune simulator server to predict the primary and secondary response of the immune system to three times of injection of the candidate vaccine. From cytokines simulation plot, we noted an increase in amounts of IL-4 and INFγ, similar to that Huang et al.^2^ observed in clinical features of COVID-19 patients (Fig. 5J). Appropriate activation in antigen-presenting cells (APC) cells, the high production of memory cells due to the extensive activation of B-cells and T-cells, control and clearance of antigens due to the creation of cytokines by the participation of TH memory cells and the evident long-term memory persistence after three times of injection, could confirm the efficiency of our candidate vaccine^44^.

## Conclusion

The goal of this research was to suggest a computational method for predicting protective B-cell and T-cell epitopes of the Envelope (E) protein of SARS-CoV-2 to construct a chimeric peptide candidate against this pandemic disease. The results of the present study demonstrated a high affinity of this chimeric peptide to MHC molecules of the immune system, and the outputs of immune response simulation to the injection of this novel vaccine confirmed our findings. To conclude, the multi-epitope vaccine designed against E protein of SARS-CoV-2 by utilizing immunoinformatics methods may be considered as a new, safe, and efficient approach against SARS-CoV-2.

## Methods

### Retrieval protein sequence

Based on vaxquery database (http://www.violinet.org/vaxquery/) envelope (E) protein of SARS- CoV-2 can be a target to design vaccines because there is a vaccine in research status working on this protein. The amino acid sequence of the envelope (E) protein and spike protein of SARS- CoV-2 were collected from the NCBI virus (https://www.ncbi.nlm.nih.gov/labs/virus/vssi/#/) with the accession number of QHD43418 and QHR63280, respectively.

### B-cell epitopes prediction

B-cell epitopes are pieces of proteins or other molecules in which antibodies (produced by B- cells) bind. Prediction methods are both time saving and cost-effective, and reliable approaches for predicting linear B-cell epitopes thus would be the first move in leading the genome-wide quest for B-cell antigens in pathogenic organism^45^ (Tab. 1). ABCpred database (http://crdd.osdd.net/raghava/abcpred/) has analyzed the full-length envelope (E) sequence for the prediction of linear B-cell epitopes. ABCpred uses a machine learning methodology that requires fixed-length patterns for training or research, while B-cell epitopes range from 5 to 30 residues. To overcome this issue, the server sought to create data sets of fixed-length patterns from B-cell epitopes by removing or linking residues to terminals. (http://crdd.osdd.net/raghava/abcpred/).

### T-cell epitopes prediction

T-cell epitopes are a group of peptides that can be detected by T-cell receptors after a given antigen have been processed intracellularly and attached to at least one MHC molecule and expressed on the surface of the APC as an MHC-peptide complex. Entities that have at least one MHC molecule that can most eagerly attach to allergenic amino acid sequences from an allergen and at the same time have the correct T-cell clone that can detect this MHC-peptide complex are known to be genetically susceptible to allergic reactions to this allergen. This concept can be investigated in in-silico by employing advanced statistical methods focused on sophisticated mathematics and statistics^46^. The first group of T-cell epitope prediction was performed using vaxign server (http://www.violinet.org/vaxign/) (Tab. 1). Vaxign is the first web-based vaccine design program to predict vaccine targets relying on genome sequences using the reverse vaccine strategy. Foreseen features of the Vaxign pipeline provide protein subcellular location, transmembrane helices, adhesion probability, human or mouse protein retention, sequencing exclusion from the genome(s) of non-pathogenic strain(s), and epitope binding to MHC molecules^47^. The next two groups helper T lymphocyte (HTL) epitopes and cytotoxic T lymphocytes (CTL) epitopes were predicted individually by the IEDB database (http://tools.iedb.org/mhcii/ and http://tools.iedb.org/mhci/) (Tab 1). There is a tool available on the IEDB database to predict binding epitopes to MHC class I molecules. This device can take a series of amino acids, or sequences, and assess the capacity of each subsequence to bind to a different MHC class I molecule. (http://tools.iedb.org/main/tcell/). The other tool that is available on the IEDB database estimates peptide attachment to MHC class II molecules. This tool uses a variety of methods to predict MHC class II epitopes, including a consensus approach combining NN-align, SMM-align, and combinatorial library methods (http://tools.iedb.org/main/tcell/).

### Antigenicity, allergenicity and toxicity prediction

VaxiJen v2.0 (http://www.ddg-pharmfac.net/vaxijen/VaxiJen/VaxiJen.html) with a threshold of 0.4 was used to predict the antigenicity of both B-cell and T-cell epitopes (Tab. 1). A threshold of 0.4 was used to predict the antigenicity of both B-cell and T-cell epitopes. VaxiJen is the first alignment-independent antigen predictor server. It is created to make the categorization of antigens solely based on the physicochemical properties of proteins without recourse to sequence alignment. The system can be used either on its own or in conjunction with alignment-based prediction methods^48^. VaxiJen v2.0 was used to estimate the antigenicity of whole peptide chimera. Based on this server, the antigenicity score of final protein was 0.5841 (Probable ANTIGEN) with 0.4 thresholds. Likewise, ANTIGENpro (http://scratch.proteomics.ics.uci.edu/) was utilized to predict the antigenicity of peptide chimera. ANTIGENpro is an alignment-free, sequence-based, and pathogen-independent protein antigenicity predictor. The forecasts are a two-stage model based on multiple versions of the primary sequence with five machine learning algorithms. The final SVM classifier analyses the corresponding predictions and determines whether or not the protein is probable to be antigenic or not, as well as the relevant probability. ANTIGENpro is the first indicator of all protein antigenicity trained to employ reactivity data from the protein microarray analysis of five pathogens. (http://scratch.proteomics.ics.uci.edu/explanation.html#ANTIGENpro) AllerTOP v2.0 (http://www.ddg-pharmfac.net/AllerTOP/feedback.py) was used to predict the Allergenicity of both B-cell and T-cell epitopes (Tab. 1). Protein sequences are sent to this server in simple text. The results page shows the identity of an allergen: “Probable Allergen” or “Probable Non-allergen”. The whole protein chimera was predicted as a “probable non-allergen” using this tool^49^.

ToxinPred (https://webs.iiitd.edu.in/raghava/toxinpred/protein.php) with a peptide fragment length of 10 was used to predict toxicity of both B- and T-cell epitopes (Tab. 1). ToxinPred is a computational tool built to anticipate and design toxic/non-toxic peptides. The primary dataset used for this approach is comprised of 1805 toxic peptides (≤ 35 residues) (http://crdd.osdd.net/raghava/toxinpred/). This server also was used to predict the toxicity of whole protein chimera and no fragment was predicted as toxin.

### Construction of chimeric peptide

Selected B-cell and T-cell epitopes (Tab. 1) were fused to construct the protein chimera as a multi-epitope vaccine. Overlaps of B-cell and T-cell epitopes were merged. KK linkers (flexible linkers) were used to connect the epitopes. The bi-lysine (KK) linker was implanted between separate epitopes to maintain their independent immunological functions (Fig. 1). KK is the target sequence of cathepsin B, which is one of the essential antigen processing proteases in the sense of the MHC class II antigen presentation^31^. The CTB subunit was chosen as an adjuvant and applied to the amino terminals of the multi-epitope peptide. There are several clear advantages to the use of adjuvants: 1) stabilizing the formulation of the vaccine, since physical structure of immunostimulators makes them highly unstable in aqueous solutions; 2) co-location of the vaccine antigen and adjuvant, assuring the activation of the same cells that have faced the antigen^50^. PAPAP linkers as rigid linkers were used to link adjuvants to epitopes (Fig. 1) in order to improve biological activity^32^.

### Amino acid composition and physicochemical properties and solubility prediction

Protparam database (https://web.expasy.org/protparam/) was used to calculate and predict the molecular weight, isoelectronic point value (PI), in vivo, and in vitro half-life, instability index II and grand average of hydropathicity (GRAVY). ProtParam from the ExPASy server is a reliable algorithm to compute Physicochemical properties. However, it uses a single sequence per analysis through the interface.

The Iupred2a server (https://iupred2a.elte.hu/) was used to analyzing disorders which make the final protein unstable (Fig. 2A). The structural states of proteins involve organized globular domains as well as fundamentally distorted protein areas that function as extremely variable conformational ensembles in isolation. IUPred2A is an integrated web interface that produces energy estimation dependent on IUPred2 order and disordered residue predictions and abnormal binding regions by ANCHOR2. The application produces visual and text outputs^51^.

SOLpro from ANTIGENpro (http://scratch.proteomics.ics.uci.edu/explanation.html#SOLpro) was used to predict the solubility of peptide chimerA upon overexpression. SOLpro predicts the tendency of the protein to be soluble when overexpressed in E.coli using a two-stage SVM model based on multiple representations of the primary sequence. Each first layer classifier uses a separate set of features describing the sequence as input. The final SVM classifier sums up the resulting estimates and predicts whether the protein is soluble or not, as well as the relevant probability. (http://scratch.proteomics.ics.uci.edu/explanation.html#SOLpro).

PepCalc server (https://pepcalc.com/) is another server to predict the solubility of the final protein. It is only a very rough estimation of water solubility (https://pepcalc.com/).

### Secondary structure prediction

Prabi server (https://prabi.ibcp.fr/htm/site/web/services/secondaryStructurePrediction) was used to predict the secondary structure of the final sequence of peptide chimera (Fig. 2B). All PRABI components provide services in their various areas of expertise (e.g., molecular, phylogeny, genomics, transcriptomics, proteomics, protein structure, and medical biostatistics (http://www.prabi.fr/spip.php?page=services).

PSIPRED 4.0 (http://bioinf.cs.ucl.ac.uk/psipred/) is another severs to predict secondary structure employed to achieve the details of residue’s configurations. It is a very simple system of secondary prediction based on a simple neural network evaluation of PSI-BLAST-generated pro les and is capable of generating findings that place the process at the very top of the current forecasting system crop^52^.

### Tertiary structure homology modelling

I-TASSER server (https://zhanglab.ccmb.med.umich.edu/I-TASSER/) was used to carry out the homology modeling of the final protein (Fig. 3A). I-TASSER is a hierarchical method for protein structure and function forecasting. First, it identifies structural PDB templates, with full-length atomic models built through iterative template-based fragment assembly simulations. After the structural integration simulation, I-TASSER uses the TM-align structural alignment software to match the first I-TASSER configuration to all the structures in the PDB database. Then it reports the top 10 proteins from the PDB that have the closest structural similarity^53–55^. UCSF Chimera was used to create a high quality image to present the predicted model. UCSF Chimera is a visualization tool to analysis the molecular structures^56^.

### Tertiary structure refinement

GalaxyWEB server (http://galaxy.seoklab.org/index.html) was used to refine the tertiary structure predicted model. In this refinement process, the server first reconstructs side chains and performs side-chain repacking following eventual overall structure relaxation through molecular dynamic simulation^40,57^.

### Validation of the tertiary structure

Model validation is an essential step in the model construction process as it identifies possible defects in the 3D structures expected^58^.Ramachandran plot analysis was performed using the Procheck server (https://servicesn.mbi.ucla.edu/PROCHECK/ ) (Fig. 3D); as a program to control the stereochemical consistency of the protein structure (https://www.ebi.ac.uk/thornton- srv/software/PROCHECK/). The Ramachandran plot is particularly useful as a test for geometric validation since φ and ψ are not part of the target function for refining. The percentage of residues found in the most favored φ and ψ regions is strongly correlated with resolution and is now reported as standard in protein structure papers, whereas specific “outlier” residues incorrect structures are used to denote either possible errors or possibly significant strained conformations^59^. ProSA-web (https://prosa.services.came.sbg.ac.at/prosa.php) was used to analyze the Z-score of the 3D model predicted (Fig. 3C). The Protein Structure Analysis (ProSA) program is a well-established method with a large user base. It is widely used in the optimization and validation of experimental protein structures and structural prediction and simulation^60^. The Z-score shows the overall quality of the model and calculates the divergence of the total energy of the structure from the distribution of energy from spontaneous conformations^61^.

### Molecular docking of subunit vaccine with MHC molecules

PEP-FOLD 2.0 from the RPBS Web Portal server (https://mobyle.rpbs.univ-paris-diderot.fr/cgi-bin/portal.py#forms::PEP-FOLD) performed the prediction of the tertiary structure of vaccine construct epitopes. PEP-FOLD is an online tool that is designed to model 3D peptide conformation structures in aqueous solutions for 9–25 amino acid length peptides (*de novo* modeling). PEP-FOLD conducts a series of 50 simulations, beginning with an amino acid sequence and returns the most critical energy and population-related conformations to be found^62^.

The ClusPro 2.0 server rotates each Final epitopes ligand and whole vaccine peptide with 70,000 rotations. It translated the ligand rotations relative to the MHC receptor alleles in 3 axes (x,y,z) on a grid. Then, it chose the scores of top 1000 lowest energy docked structures 70,000 rotations. These 1000 lowest energy docked structures would be processed subsequently. This set might have the potential to consist of at least some models which are close to the native structure of the complex. Then, the server clustered the 1000 rotations by finding the structure with the most “neighbors” within 9 Å IRMSD radios as the distance measure. Then, it considered this ligand and its neighbors as the “cluster center” and the “members” of the cluster, respectively. This process was repeated under remainder of the ligands to find the next clusters. Finally, the server scores the models and reported the top score ones based on the cluster size (10 most populated clusters)^24,25^.

PyMOL software was used to analyze docking results. PyMOL is mostly utilized for molecular visualization by crystallographic, molecular dynamic simulation, and protein modeling software packages^63^.

### Immune response simulation

IL4, IL10 and INFγ inducing peptides from the 6 epitopes in the final vaccine construction were predicted via IL4pred server (https://webs.iiitd.edu.in/raghava/il4pred/design.php), IL-10Pred server (https://webs.iiitd.edu.in/raghava/il10pred/predict3.php) and INFepitope server (https://webs.iiitd.edu.in/raghava/ifnepitope/predict.php), respectively.

Immune response to vaccine injection was simulated by the C-IMMSIM server (http://150.146.2.1/C-IMMSIM/index.php) (Fig. 5). C-ImmSim is an agent-based computational immune response simulator that utilizes position-specific score matrix (PSSM) and machine learning methods for predicting epitope and immune interactions, respectively^64^. We regulate the parameters based on the predominant HLA alleles of predictions. The host HLA selection parameter for MHC class I was set on A1010, A1101, and B0702 and for DR MHC class II was sat on DBR1_0101 and based on literatures the time step to injection parameter was set on 1, 84 and 100 (maximum allowed value), respectively. We randomly shuffled the vaccine protein sequence (without adjuvants) by using the Stothard P (2000), the Sequence Manipulation Suite server (https://www.bioinformatics.org/sms2/shue_protein.html)^65^ to create a control group.

The immune system simulation server mentioned here provides an opportunity to study the overall immunogenicity of the generic protein sequence in the context of its amino acid sequence^66^. The total simulation is focused on three events: 1) B-cell epitopes binding, 2) HLA class I and II epitopes binding, and 3) TCR binding, which HLA-peptide complex interaction should be presented. Such processes are independently conducted by cells described by agents and occupy a specified simulated biological amount^66^.

## Declarations

## Figures and Tables

**Table 1:** T-cell and B-cell protective epitopes

Epitope	B-cell	MHC I	MHC II	Antigenicity	Allergenisity	Toxicity
**ABCpred**						
○ TLAILTALRLCAYCCN	+*	+	−**	Antigen	Non-Allergen	Toxin
√ NVSLVKPSFYVYSRVK	+	−	−	Antigen	Non-Allergen	Non-Toxin
√ YVYSRVKNLNSSRVPD	+	+	+	Antigen	Non-Allergen	Non-Toxin
○ LCAYCCNIVNVSLVKP	+	−	−	Antigen	Non-Allergen	Toxin
○ FVSEETGTLIVNSVLL	+	−	−	Non-Antigen	Discontinued	Discontinue
**IEDB database**						
√ SRVKNLNSSR	−	+	−	Antigen	Non-Allergen	Non-Toxin
√ SFVSEETGT	−	+	−	Antigen	Non-Allergen	Non-Toxin
√ SRVKNLNSS	−	+	−	Antigen	Non-Allergen	Non-Toxin
√ VYSRVKNLNS	−	+	−	Antigen	Non-Allergen	Non-Toxin
√ VYSRVKNLN	−	+	−	Antigen	Non-Allergen	Non-Toxin
√ RVKNLNSSR	−	+	−	Antigen	Non-Allergen	Non-Toxin
√ SFYVYSRVKNLNSSR	−	−	+	Antigen	Non-Allergen	Non-Toxin
√ FYVYSRVKNLNSSRV	−	−	+	Antigen	Non-Allergen	Non-Toxin
√ YVYSRVKNLNSSRVP	−	−	+	Antigen	Non-Allergen	Non-Toxin
○ IVNSVLLFLAFVVFL	−	−	+	Antigen	Allergen	Discontinue
√ FYVYSRVKNLNSSRV	−	−	+	Antigen	Non-Allergen	Non-Toxin
√ SFYVYSRVKNLNSSR	−	−	+	Antigen	Non-Allergen	Non-Toxin
**Vaxign server**						
○ LVKPSFYVY	−	+	+	Antigen	Allergen	Toxin
○ SLVKPSFYV	−	+	+	Antigen	Allergen	Toxin
√ RVKNLNSSR	−	+	−	Antigen	Non-Allergen	Non-Toxin
○ SLVKPSFYVY	−	+	−	Non-Antigen	Discontinued	Discontinue
√ YVYSRVKNL	−	+	−	Antigen	Non-Allergen	Non-Toxin
√ VTLAILTAL	−	+	−	Antigen	Non-Allergen	Non-Toxin
√ TLAILTALR	−	+	−	Antigen	Non-Allergen	Non-Toxin
√ VTLAILTA	−	−	+	Antigen	Non-Allergen	Non-Toxin
○ YSRVKNLNS	−	−	+	Antigen	Allergen	Toxin

T-cell epitopes were identified as best epitopes based on number of alleles and antigenicity score.

* Related

** Unrelated

√ Selected epitopes for vaccine construction

○ Unselected epitopes for vaccine construction.

**Table 2:** Docking results

_Vaccine Epitopes_╲^ Weighted Score of docked complex with ^	HLA-A*0l:01	HLA-A*24:02	HLA-B*07:02	HLA-A*11:01	HLA-A*03:01	HLA-DPA1*01:03	HLA-DRB1*01:01
**VTLAILTAL**	−732.3	−765.2	−662.7	−644.7	−682	-------	-------
**TLAILTALR**	−700	−626.2	−587.3	−601.5	−635.8	-------	-------
**SFVSEETGT**	−650.2	−626.4	−523.8	−548.9	−503.3	-------	-------
**LVTLAILTA**	−777.3	−777.3	−641.3	−660.8	−682	-------	-------
**NVSLVKPSFYVYSRVK**	-------	-------	-------	-------	-------	−859.9	−972.9
**SFYVYSRVKNLNSSRVPD**	-------	-------	-------	-------	-------	−959.7	−998.1

The weighted scores of the lowest energy docked structures were based on the cluster size of the most populated cluster.
